# Multiscale investigation on the chemical and anatomical changes of lignocellulosic biomass for different severities of hydrothermal treatment

**DOI:** 10.1038/s41598-021-87928-y

**Published:** 2021-04-19

**Authors:** Julia P. Lancha, Patrick Perré, Julien Colin, Pin Lv, Nathalie Ruscassier, Giana Almeida

**Affiliations:** 1 CentraleSupélec, Laboratoire de Génie des Procédés et Matériaux, SFR Condorcet FR CNRS 3417, Centre Européen de Biotechnologie et de Bioéconomie (CEBB), Université Paris-Saclay, 51110 Pomacle, France; 2 CentraleSupélec, Laboratoire de Génie des Procédés et Matériaux, Université Paris-Saclay, 8-10 rue Joliot-Curie, 91190 Gif-sur-Yvette, France; 3 INRAE, AgroParisTech, UMR SayFood, Université Paris-Saclay, 91300 Massy, France

**Keywords:** Biofuels, Bioalcohols

## Abstract

The chemical changes sustained by lignocellulosic biomass during hydrothermal treatment are reflected at multiple scales. This study proposes to benefit from this multiscale nature in order to provide a global understanding of biomass alterations during hydrothermal treatment. For this purpose, complementary imaging techniques—confocal Raman microscopy and X-ray nano-tomography—analysed by image processing and coupled to chemical measurements were used. This unique combination of analyses provided valuable information on topochemical and morphological changes of poplar samples, without the artefacts of sample preparation. At the cell wall level, holocellulose hydrolysis and lignin modifications were observed, which corresponded to anatomical modifications observed at higher scales. Overall, after treatment, samples shrank and had thinner cell walls. When subjected to more severe pre-treatments, cells were disrupted and detached from adjacent cells. Anatomical changes were then used to obtain quantitative indicators of the treatment severity. The effects of treatment at different scales can thus be quantitatively connected in both directions, from micro to macro and from macro to micro.

## Introduction

Most biological materials have hierarchically organized structures whose properties are affected by their chemical composition, the interactions between components, and by their spatial distribution and organization. These relationships between properties are observed for lignocellulosic biomass and, particularly, wood.

Wood cell walls are composed of layers that differ in their structure, thickness and composition. The main layers are the primary cell wall and the secondary cell wall, which is composed of three layers (S1, S2 and S3). The structure between two adjacent cells is called the middle lamella, but due to the difficulty in distinguishing the primary cell wall from the middle lamella, they are often analysed together as the compound middle lamella^1^. In this study, we focus on two of these layers: the secondary cell wall, easier to observe because of its large thickness; and the compound middle lamella, because it ensures bonding between neighbour cells. Even though each layer has distinct characteristics, they are all composed primarily of three polymers: cellulose, hemicellulose and lignin.

Cellulose is the main component of wood, accounting for 45–47% and 40–45% of hardwood and softwood weight, respectively^2^. It is a polymer of β-D-glucopyranose^1^ linked by 1,4-glycosidic bonds^3^. Its linear nature and the presence of multiple hydroxyl groups^3^ make it likely to form intra- and intermolecular hydrogen bonds. This chemical feature has structural consequences: cellulose is highly organized into microfibrils^4^, forming a crystalline core with a semi-crystalline shell. This structure gives wood its strength. Moreover, the crystalline nature of cellulose also makes it resistant to chemical attack^1^.

Unlike cellulose, hemicellulose is a type of polymer composed of different pentose and hexose monosaccharides^5^. In hardwoods, hemicelluloses constitute 25–40% of wood by weight^2^ and are mainly composed of glucuronoxylans, which consist of a xylose unit backbone with 4-O-methylglucoronic acid and O-acetyl side groups. In softwoods, they can constitute 25–29% of wood by weight^2^ and have glucomannan as their main constituent^6^. Their lower degree of polymerization and branched structure make them more sensitive to temperature and chemical attacks than cellulose^1^.

Lignins are heterogeneous, high molecular weight phenolic compounds that constitute 20–25% and 30–60% of hardwoods and softwoods, respectively^2^. They are composed of three basic units: syringyl (S), guaiacyl (G) and p-hydroxyphenol (H) phenylpropanoid moieties. Lignins frequently form covalent bonds with surrounding carbohydrates, especially hemicelluloses^3^. Softwood lignins are almost entirely composed of G units, while hardwoods have both S and G units^7^.

These three components form an extremely recalcitrant matrix, requiring pre-treatment when lignocellulosic biomass is utilized in the production of bioethanol. The main goal of the pre-treatment step is to favour the following enzymatic hydrolysis by improving the accessibility of polysaccharides, which increases the yield of fermentable sugars^8^. Among the vast range of pre-treatments available, steam explosion is notable as it requires no additional solvent and can be applied to a variety of raw materials. It consists of two steps, the first of which is hydrothermal treatment, during which biomass is subjected to temperatures up to 230 °C under steam-saturated pressure for several minutes^9^. The pressure is then rapidly released in the explosion step. The changes experienced by biomass during hydrothermal treatment greatly influence the explosion and the subsequent bioethanol production steps and impact the efficiency of the entire production chain.

A full understanding of the effects of pre-treatment on lignocellulosic biomass, including both chemical and structural aspects, can only be achieved through a multiscale approach. From a molecular point of view, partial hydrolysis releases mono- and oligosaccharides, which has been extensively demonstrated in literature^4,10^. At larger scales, cell wall breakdown can alter mechanical and anatomical biomass properties. Despite the effect of structural changes on the subsequent enzyme accessibility, very little attention has been paid to the cell wall and cell scales^11,12^. This fact probably arises from the difficult sample preparation processes required to, at the same time, expose anatomical features of interest—i.e. different cell wall layers—while preserving the original structure of the sample^13^. Combining several imaging techniques can be a powerful tool in addressing this issue. Raman spectroscopy is of particular interest because it provides a chemical fingerprint of the material and, combined with microscopy, allows the assessment of sub-parietal composition. It has proven useful mainly in the study of lignocellulosics^14,15^. One disadvantage of using confocal Raman microscopy is that sample preparation requires cutting the sample into thin layers, which can alter its morphology. In contrast, X-ray nano-tomography is non-destructive, allowing morphological assessment of the same sample before and after treatment. The results obtained from this technique provide morphological information on the tissue scale with a sub-micrometric resolution. For the first time in this field, we combine these two imaging techniques to characterize hydrothermal treatment by providing a global, multiscale view of biomass changes during the process.

## Results and discussion

### Chemical modifications

**Figure 1:**
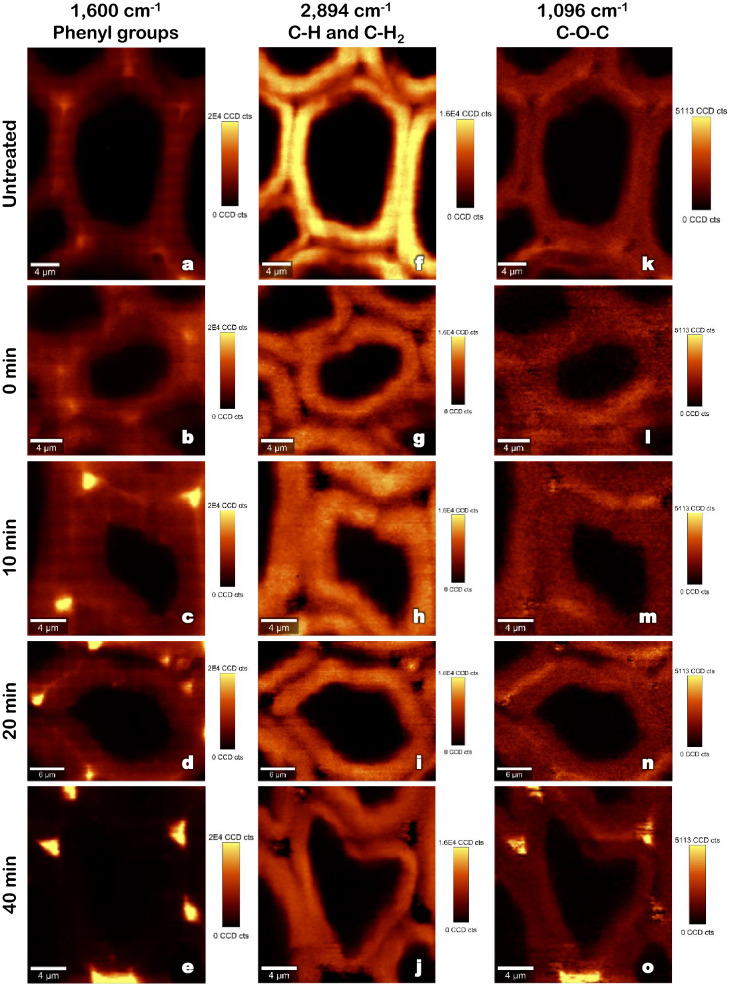
Raman images obtained by integration at different wavenumbers of untreated and hydrothermally treated poplar samples (160 °C) [Images were obtained with the help of Project FOUR software (Witec)].

**Figure 2:**
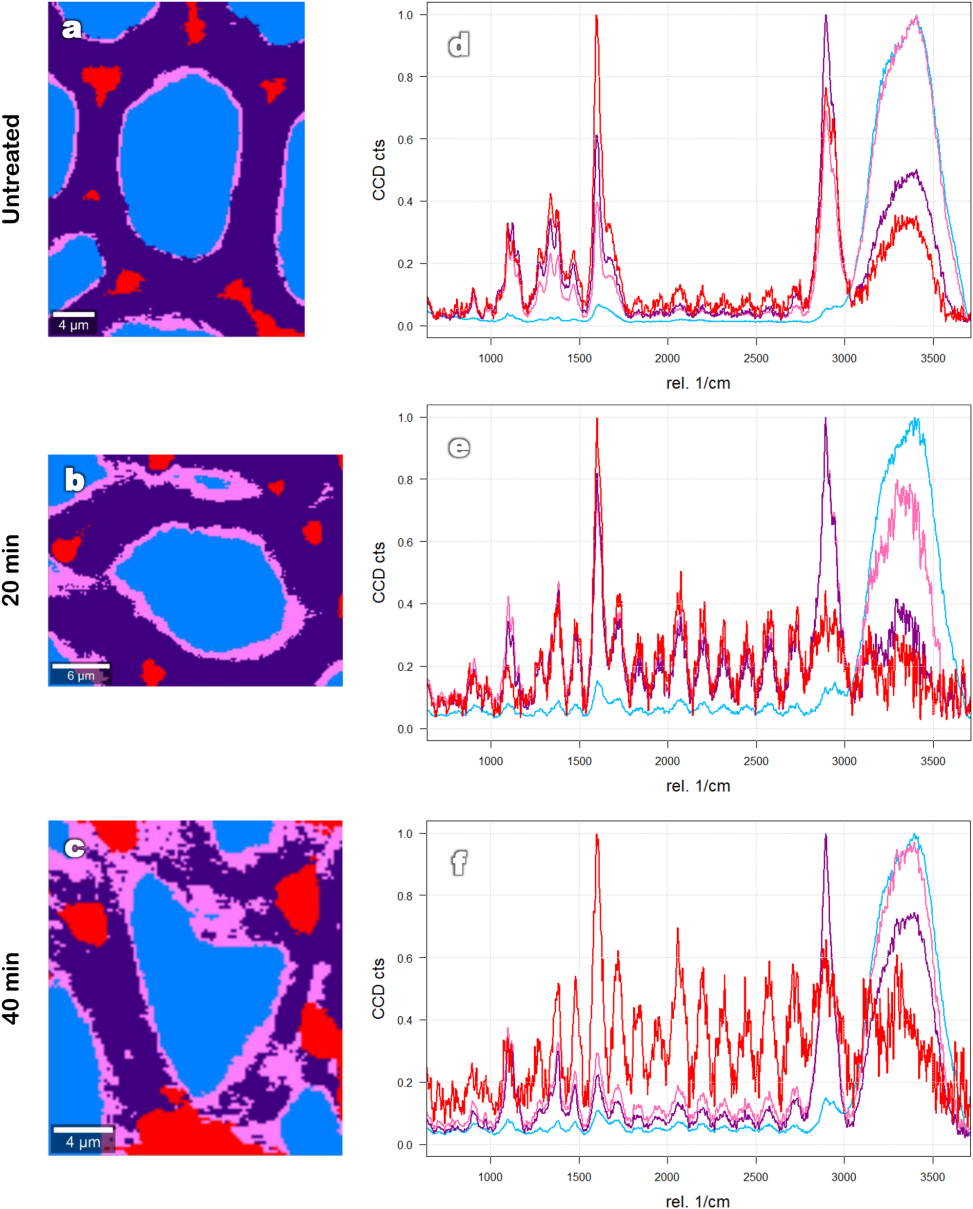
Raman images of untreated (**a**) and hydrothermally treated (160 °C) poplar samples based on hierarchical cluster analysis (**b**,**c**) and the average spectra of respective clusters (normalized) (**d**,**f**). [Images (**a**–**c**) were obtained with the help of Project FOUR software (Witec)].

**Figure 3:**
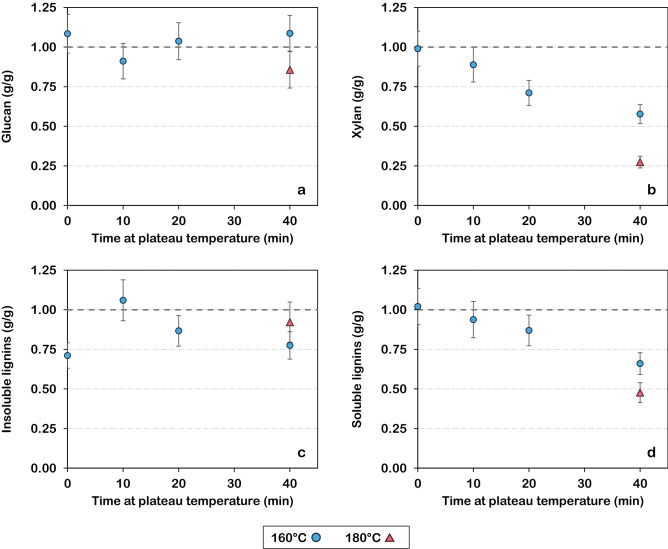
Relative amounts of (**a**) glucan, (**b**) xylan, (**c**) insoluble lignins and (**d**) soluble lignins of hydrothermally treated poplar as compared to its native state.

Figure 1 presents the results obtained by confocal Raman microscopy after univariate analysis for a native sample and for samples treated at 160 °C for 0, 10, 20 and 40 min. The same samples had their data submitted to a cluster analysis. Only the most severely treated samples (20 and 40 min) were chosen to be represented in Fig. 2, due to their higher contrast with the native sample. Figure 2 also provides the respective average spectra of the clusters, normalized with the maximum value. Finally, results obtained by Raman confocal microscopy (Figs. 1 and 2) have been compared with the chemical composition of hydrothermally treated biomass (Fig. 3).

In Raman spectrometry, lignins are expected to exhibit an intense peak at 1600 cm^-1^, attributable to phenyl groups of lignins^16^ and a second peak at approximately 1650 cm^-1^, that corresponds to the ring conjugated C=C stretching of the coniferaldehyde and coniferyl alcohol units. It is less intense and usually observed as a shoulder of the first one^15,17^.

Analysing hemicelluloses and cellulose through this technique is more challenging than lignins. Due to their similar structure, several bands appear at the same wavenumber^16^, making it impossible to distinguish hemicelluloses from cellulose. They are thus analysed together, as holocellulose. The band at approximately 2894 cm^−1^ corresponds to the aliphatic C–H and C–H_2_ stretch from carbohydrates^16,17^ and is commonly used for the study of wood carbohydrates due to a good signal-to-noise ratio^15^.

#### Lignins

To obtain chemical maps for lignins, peaks at 1600 cm^−1^ and 1650 cm^−1^ were integrated together as it resulted in a better signal-to-noise ratio and did not alter the determination of lignins distribution^15^. Figure 1a shows the intensity of this broad band in an untreated wood cell. It can be seen that, even though lignins are present in all regions of the cell wall, signal intensity is higher (seen in yellow and orange) in regions corresponding to the cell corner and the middle lamella. These results are consistent with those of previous studies^15,17^.

After treatment (Fig. 1b–e), it was possible to observe a reduction of signal intensity in zones corresponding to the secondary cell wall. At the same time, the signal seemed to intensify in the intercellular regions, such as the triple points and middle lamella. This effect intensified with increasing treatment time and became particularly evident in the spectra obtained by cluster analysis (Fig. 2). In the untreated sample, regions corresponding to cell corners (red) presented a clear predominance of the lignins band (1600 cm^−1^). After 20 and 40 min at the temperature plateau, holocellulose signal intensity (2894 cm^−1^) decreased in comparison with lignins in the cell corners. On the other hand, the contribution of lignins to the spectra of the secondary cell wall (purple) became weaker as the treatment time increased.

Based on these results, it is possible to assume that different cell wall regions responded differently to hydrothermal treatment. An increase in lignin signal intensity in high lignified cell wall regions has also been observed during dilute acid pre-treatment of *Pinus bungeana* Zucc^18^. A few hypotheses may explain mechanisms behind the observed changes, likely the result of a combination of different factors.

Some authors consider solubilization one of the modifications experienced by lignins in hydrothermal treatments^10^. The acidic condition leads to the breaking of lignin—carbohydrate bonds and later, to depolymerization of lignins^19^. Solid biomass would then suffer a loss of lignins to the treatment liquid, which could explain the reduction of signal intensity observed on the secondary cell wall. This hypothesis is supported by a reduction of the lignin content in the solid fraction after treatment, as shown in Fig. 3. This behaviour is observed for both acid soluble (Fig. 3d) and acid insoluble (Fig. 3c) fractions of lignins, for which 34 and 22% of the respective initial amounts are lost after 40 min of treatment at 160 °C. Additionally, the monomeric composition of lignins influences its susceptibility to hydrothermal degradation. Syringil (S) units present fewer cross-links, which makes it more susceptible to hydrothermal treatment^10,20^. The predominance of S units in the secondary cell wall^20^ is one possible cause of the differential attack observed in Raman images. Using paired samples, a previous work has demonstrated by in situ viscoelastic measurements and FTIR that lignin depolymerization prevails during hydrothermal treatment at 160 °C until 30 min of temperature plateau^21^. However, it only explains the decrease in the signal intensity in the secondary cell wall, but not its increase in other regions.

**Figure 4:**
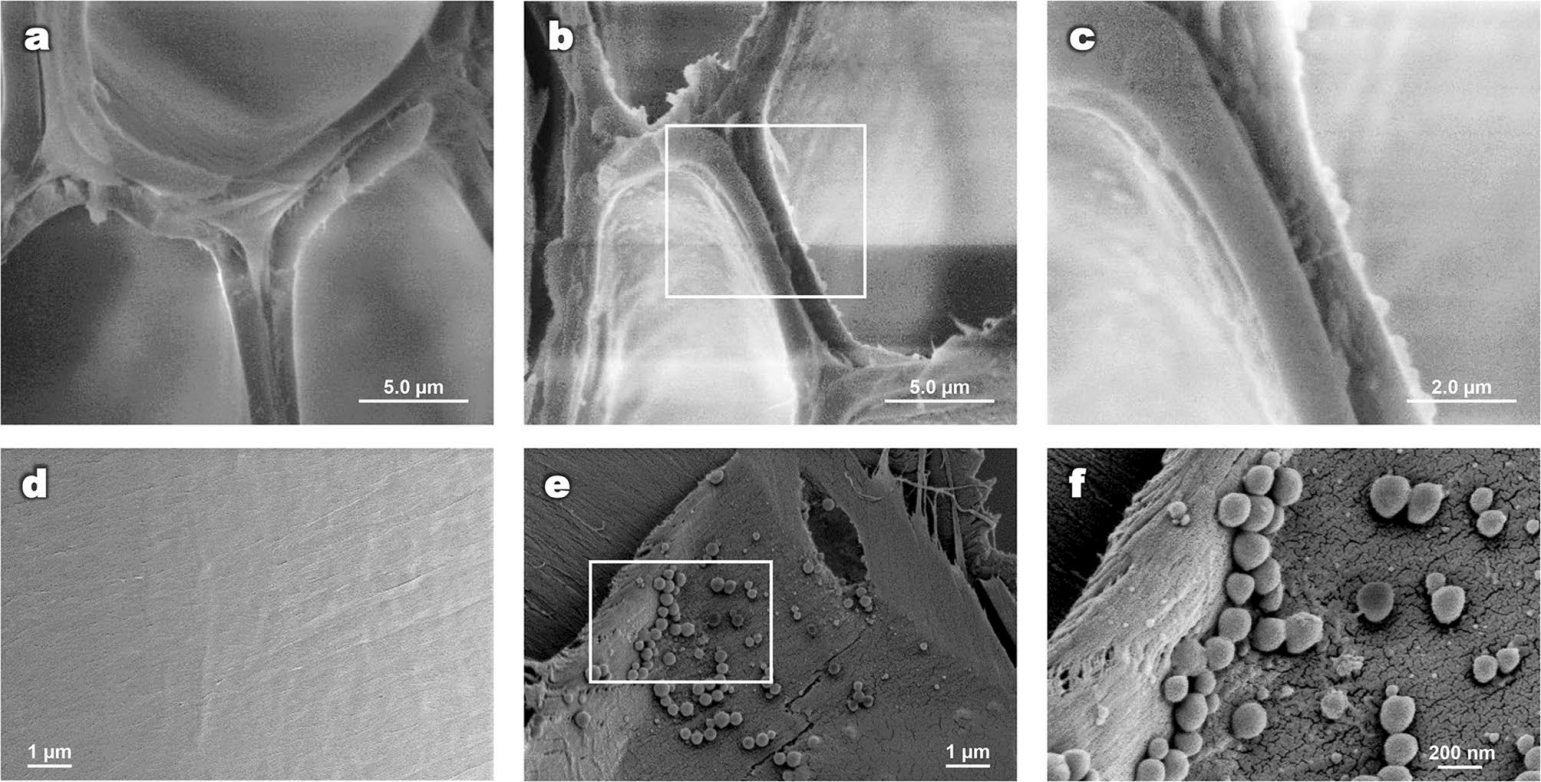
SEM (**a**–**c**) and FEG-SEM (**d**–**f**) images of poplar cell walls. Images before hydrothermal treatment (**a**,**d**). Images after 40 min of hydrothermal treatment at 180 °C (**b**–**e**). The regions within the white rectangles are shown in (**c**,**f**) at increased magnification. It is possible to observe cell wall detachment and the formation of round droplets.

Another possible explanation is the migration of the lignins. The treatment temperatures largely exceeded lignin’s glass transition temperature (T_g_), between 50 and 100 °C for water-saturated wood^22^. These temperatures allow lignins to melt and coalesce. They are then forced out of the cell wall matrix and form droplets during the cooling phase^23^. To verify if this hypothesis applies to the samples analysed here, further observations were made by electron microscopy, the results of which are presented in Fig. 4 (obtained by ESEM and FEG-SEM). It is possible to observe the formation of round droplets in the inner surface of the cell (Fig. 4e,f) and in the intercellular region (Fig. 4b,c), which is consistent with previous results^23^. The observed droplets had a wide range of size distribution (154 ± 110 nm, 20 nm and 352 nm, for mean, minimum and maximum diameter). The larger droplet diameters are large enough for their chemical composition to influence the Raman spectra of the region in which they are deposited. However, they might not be seen as single droplets since their size is close to the lateral diffraction limit of the confocal Raman microscope. It is then conceivable to assume that the intense lignin signal observed at triple points of pre-treated samples originate from these lignin droplets. This effect is, however, only observed at the intercellular region and not at the inner surface of the cell, where lignin droplets are also found in large quantities. This suggests that droplets found in different anatomic regions of the cell wall have different compositions and thus different contributions to the Raman spectra. Additionally, the increase observed on the content of insoluble lignins after 10 min of treatment (Fig. 3c)—exceeding by 7% the amount initially present in native biomass—proves that lignins also undergo chemical modifications, in addition to migration, affecting their solubility.

A previous study^24^ used fluorescence lifetime imaging microscopy and stimulated Raman scattering microscopy to evaluate the effect of low concentration maleic acid pre-treatment on poplar. Based on the fluorescence lifetime, they were able to distinguish two types of lignin: dense lignins and loosely packed lignins. In the untreated sample, loosely packed lignins were mainly present in the secondary wall. Dense lignins, on the other hand, were present all over the cell wall. After pre-treatment, lignins present in the cell corner formed dense lignin droplets, while lignins present in the secondary cell wall were released with hemicelluloses and end up forming loosely packed lignin droplets^24,25^. These loosely packed lignin droplets are composed of lignin-carbohydrate complexes and contain a low lignin concentration, undetectable when analysed through stimulated Raman scattering microscopy at the lignin resonance frequency (1600 cm^−1^). Therefore, our results are consistent with previous findings, showing that droplet composition depends on the anatomic region in which they are formed. This fact reinforces the importance of imaging techniques in providing a better understanding of cell wall degradation during hydrothermal treatment.

The effect of droplet formation on subsequent steps, such as enzymatic hydrolysis, remains uncertain. Lignin migration could result in higher accessibility of cellulose inside the cell wall to enzymes, but on the other hand, the presence of droplets attached to the cell wall surface could physically limit the access of enzymes or result in non-productive binding with lignins or lignin-carbohydrate complexes^26^.

Laser-induced fluorescence considerably increased with pre-treatment severity. Despite the application of photobleaching prior to the Raman analysis, a maximum was reached after 40 min of treatment (Fig. 1e). For this sample, the quality of Raman spectra (as observed by the signal-to-noise ratio) was reduced (Fig. 2f). These results thus need to be interpreted with caution, as the high signal intensity in cell corners could be overshadowing less concentrated areas. For the same reason, results obtained for more severely treated samples (such as those treated to 180 °C) through confocal Raman microscopy could not be explored and, therefore, were not presented in this study. Despite the limitations of this method, an increase in laser-induced fluorescence is also an indicator of biomass chemical modifications, as shown previously^27^. When studying lignins and lignin-derived model compounds using Raman spectroscopy, they demonstrated that freely-rotating 5–5′ linkages were a precondition for laser-induced fluorescence in wood. During treatment, more sensitive linkages (such as α- and β-aryl ether) were broken, allowing 5–5′ linkages to rotate without restrictions. These results are consistent with those of the current study and suggest a modification of lignin mobility and degree of condensation. Future studies could provide a greater understanding of the mechanisms leading to laser-induced fluorescence in treated wood and utilize it for analytical purposes.

#### Holocelluloses

Figure 1f shows the image obtained by integration over the 2894 cm^−1^ band of the Raman spectrum (corresponding to holocellulose) of an untreated poplar sample. As expected, the highest holocellulose concentration is observed in the secondary layer of the cell wall. Regions of weaker signal in this band correspond to the middle lamella and cell wall corners, which are known to have lower holocellulose concentrations^15,17^. Small intensity variations throughout the S2 region can be observed and, in addition to differences in concentration, can also be attributed to the microfibril angle of cellulose^15^. Because the chemical bonds represented by this band (C–H and C–H_2_) are perpendicular to the axis of microfibrils, the intensity of the Raman signal is maximized when microfibrils are aligned with the laser axis, orthogonal to the electric field vector.

Treated samples (Fig. 1g–j) showed a decrease in the signal intensity with an increase in treatment time. From the beginning of the temperature plateau (0 min of treatment—Fig. 1g), no influence of this band is observed in the spectra of cell corners and compound middle lamella, which appear in black. At the same time, the secondary cell wall signal intensity decreased from yellow to dark orange or red. The loss of signal intensity can be explained by the hydrolysis of hemicelluloses, which has been verified by chemical analysis (Fig. 3). After 40 min of treatment, up to 42% of the hemicelluloses (expressed in terms of the xylan content) had been hydrolysed. These results are in agreement with previous studies, where hemicelluloses have been pointed out as the major source of biomass loss during pre-treatment^4,5,28,29^. Due to a lower degree of polymerization^30^ and to a branched, non-crystalline structure, hemicelluloses are much easier to degrade than cellulose^1^. Thus, they constitute the main target of lignocellulosics pre-treatment prior to the production of second-generation biofuels. The most susceptible bonds of hemicelluloses are those of heterocyclic ether bonds^10^. Their breakage generates hemicellulose oligosaccharides and acetic acid that acts as a catalyst^10^. Hardwood hemicelluloses contain more acetyl groups than softwood, which contributes to greater hydrolysis^28^. Therefore, the signal that can still be observed for the most severe treatment (Fig. 1j) is mainly due to cellulose, as evidenced by a stable cellulose content (expressed in terms of glucan) whichever the treatment duration (Fig. 3a), whereas hemicelluloses suffer a continuous reduction (Fig. 3b).

These results are not surprising given the crystallinity of cellulose and the temperature levels applied in this study. In a previous work, FTIR analyses have been performed on paired samples^21^. It showed that the hydrothermal treatment at 160 °C has only minor impact on poplar crystallinity, even after 2.5 h, as evidenced by the reduction of only 2.2% in the hydrogen bond intensity (HBI) ratio. Other works have also shown that cellulose remains essentially unaffected during hydrothermal treatment, albeit at higher temperature levels^4,29^.

Depending on the severity of the pre-treatment, monomeric sugars released from hemicelluloses and (to a lesser degree) cellulose can undergo further dehydration, producing furfural and 5-hydroxymethylfurfural, respectively. These degradation products can later be converted into aromatic intermediates and then form pseudo-lignin^19^ through polymerization and polycondensation reactions. During the cooling process, these compounds can produce spherical structures similar to lignin droplets, which also tend to adhere to cell wall surfaces^19^. The decrease in holocellulose concentration beginning in the early stages of the hydrothermal treatment meets the necessary conditions for the production of pseudo-lignin, reinforcing the previous hypothesis that the droplets observed in Fig. 4 may not all have the same composition.

#### Cellulose

Another band—located near 1096 cm^−1^–can be used to analyse cellulose. It corresponds to the asymmetric C–O–C linkages, generally aligned with the microfibril axis^17,31^. This band is thus sensitive to cellulose orientation and can provide an idea of its supramolecular structure^31^. In the untreated sample (Fig. 1k), the intensity is evenly distributed throughout the secondary layer, with a slightly higher intensity in the S1 layer due to microfibril orientation. As expected, no signal is observed for this band in the cell corners and compound middle lamella, since these regions are essentially composed of lignin. As the treatment time increases (Fig. 1l–o), the intensity of this band increases, especially in the S1 layer. The same observation was made in a previous study^31^, in which two possible reasons to explain this signal increase were provided. Firstly, molecules may be more parallel to the beam polarization. Indeed, even though it is not completely hydrolysed during pre-treatment, cellulose undergoes a significant reduction in the degree of polymerization^32,33^. The presence of shorter polymer chains provides enhanced mobility to the cell wall, perhaps allowing more molecules to align with the laser beam. The second explanation was an increase in cellulose crystallinity, which would result either from the loss of amorphous components (hemicelluloses and amorphous cellulose)—i.e. increasing the relative amount of crystalline cellulose—or come from a genuine rearrangement of cellulose organization.

### Effect of hydrothermal treatment on the cellular morphology

**Figure 5:**
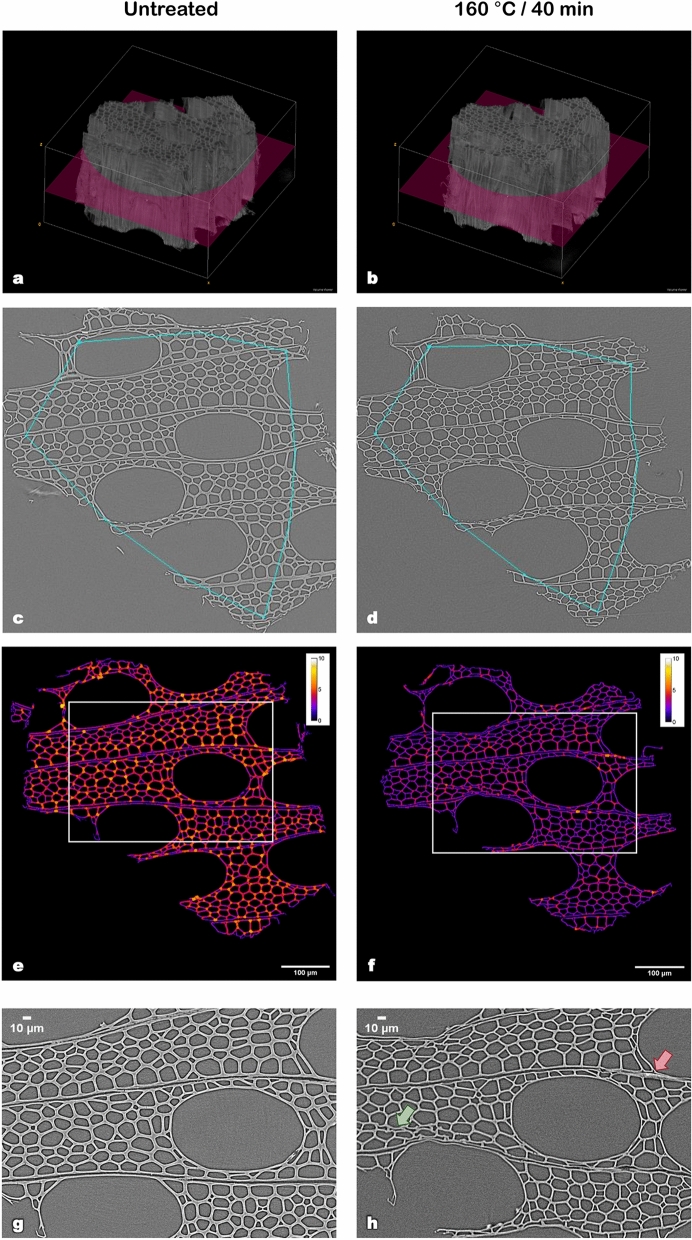
X-ray nano-tomography images of the same sample in its untreated state and after hydrothermal treatment at 160 °C for 40 min. (**a**,**b**) 3-D reconstructions of the sample, highlighting the virtual sections observed in figures (**c**–**h**) [obtained with the help of ImageJ v1.52i software; imagej.nih.gov/ij/]. (**c**,**d**) The chains of points used to determine the shrinkage of the sample. (**e**,**f**) The calculated thickness of cell walls throughout the slices (in µm) [obtained with the help of ImageJ v1.52i software; imagej.nih.gov/ij/]. (**g**,**h**) Highlights of cell wall collapse (green) and cell detachment (red), extracted from the regions within the white rectangles in (**e**,**f**).

**Figure 6:**
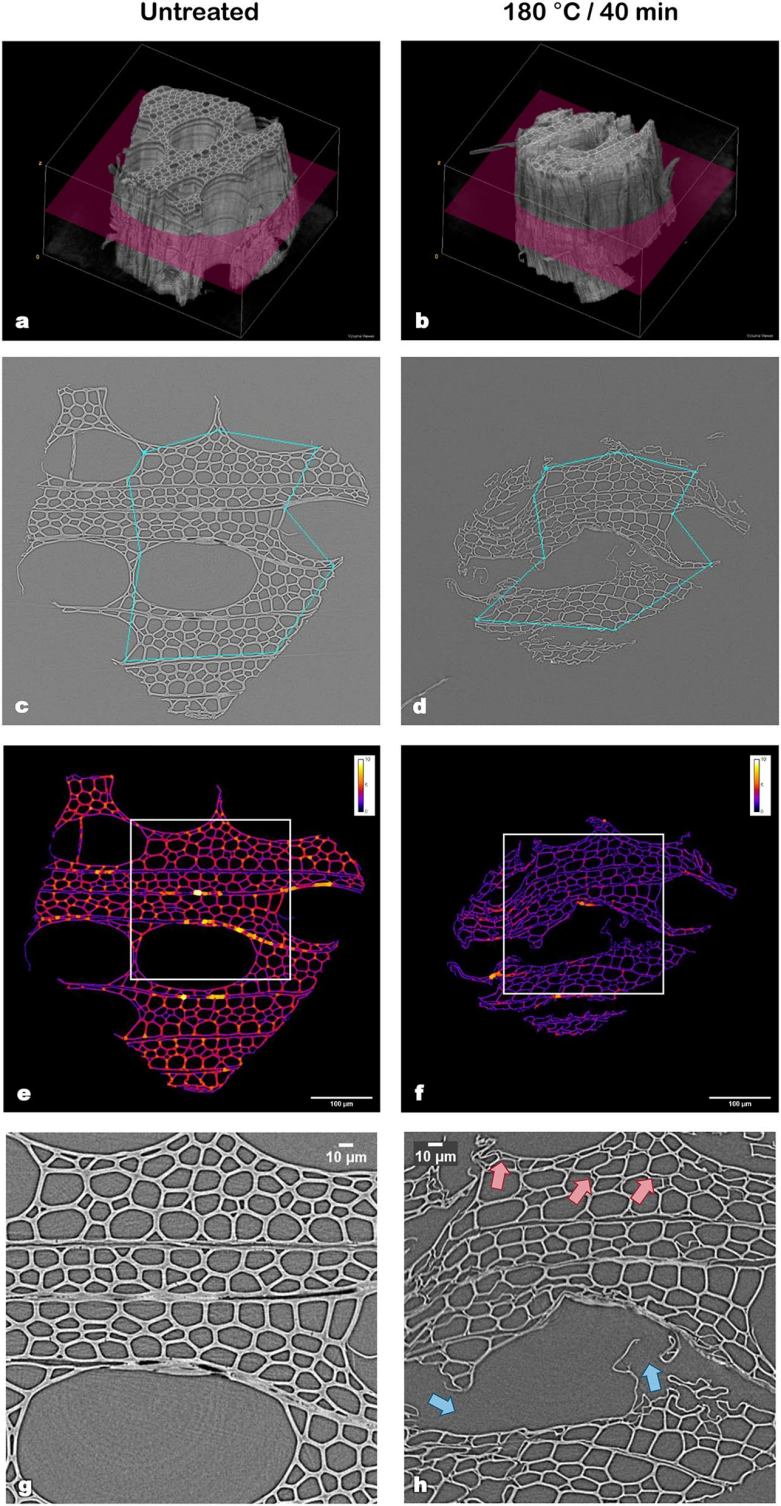
X-ray nano-tomography images of the same sample in its untreated state and after hydrothermal treatment at 180 °C for 40 min. (**a**,**b**) 3-D reconstructions of the sample, highlighting the virtual sections observed in figures (**c**–**h**) [obtained with the help of ImageJ v1.52i software; imagej.nih.gov/ij/]. (**c**,**d**) The chains of points used to determine the shrinkage of the sample. (**e**,**f**) The calculated thickness of cell walls throughout the slices (in µm) [obtained with the help of ImageJ v1.52i software; imagej.nih.gov/ij/]. (**g**,**h**) Highlights of vessel disruption (blue) and cell detachment (red), extracted from the regions within the white rectangles in (**e**,**f**).

The previous results showed the effect of hydrothermal treatment on the chemical structure of biomass cell walls. It is reasonable to presume that the changes observed at the cell wall level have an impact on the macroscopic structure of biomass. To examine this further, the anatomical structure of the samples was observed using X-ray nano-tomography. X-ray nano-tomography analyses are time-consuming. Indeed, several days are required to prepare the samples, to perform tomography and 3D reconstruction of the images—using high-performance computers. Thus, to highlight the interest of the imaging technique, only the native and most severely treated samples (40 min) for each temperature -and, therefore, those with the most significant anatomical changes- were studied. After data processing, each analysis resulted in a 3D reconstruction of the sample (Figs. 5a,b and 6a,b). It allowed for exploration of the structure before and after treatment and provided an overall picture of the modifications taking place inside the sample, without the artefacts of sample preparation. Each 3D reconstruction was later used to generate to a set of virtual cross-sectional slices, from which five slices (100 µm apart from each other) were submitted to posterior image analysis. Figures 5c–h and 6c–h show the results obtained for the central cross-sectional slice (highlighted in pink in Figs. 5a,b and 6a,b) of native and hydrothermally treated samples at 160 °C/40 min and 180 °C/40 min, respectively.

Firstly, the cell wall thickness was calculated by image processing and is represented in Figs. 5e,f and 6e,f according to the colour scale. No significant difference was found between different slices of the same sample. For both treatment temperatures, a reduction of cell wall thickness can be observed when comparing treated samples to their respective native state. The results also show that in native samples (Figs. 5e and 6e), cell wall thickness can reach up to 7 µm depending on the anatomical structure (orange–yellow colour). For pre-treated samples, it barely exceeds 5 µm in the mildest pre-treatment (red–orange colour) and 3 µm for the most severe one (pink-purple colour). The mean calculated cell wall thickness shrank from 3.8 ± 1.0 µm to 2.7 ± 0.7 µm (*p* value < 10^–6^) and from 3.5 ± 0.9 µm to 2.2 ± 0.6 µm (*p* value < 10^–11^) for 160 and 180 °C pre-treatments, respectively. It corresponded to a reduction of 29 and 36% on the cell wall thickness (for temperatures of 160 and 180 °C, respectively). A similar observation was made by electron tomography in the case of mild pyrolysis of wood^34^. Values of native cell wall thickness correspond to those found for poplar fibres in previous works^35^.

**Figure 7:**
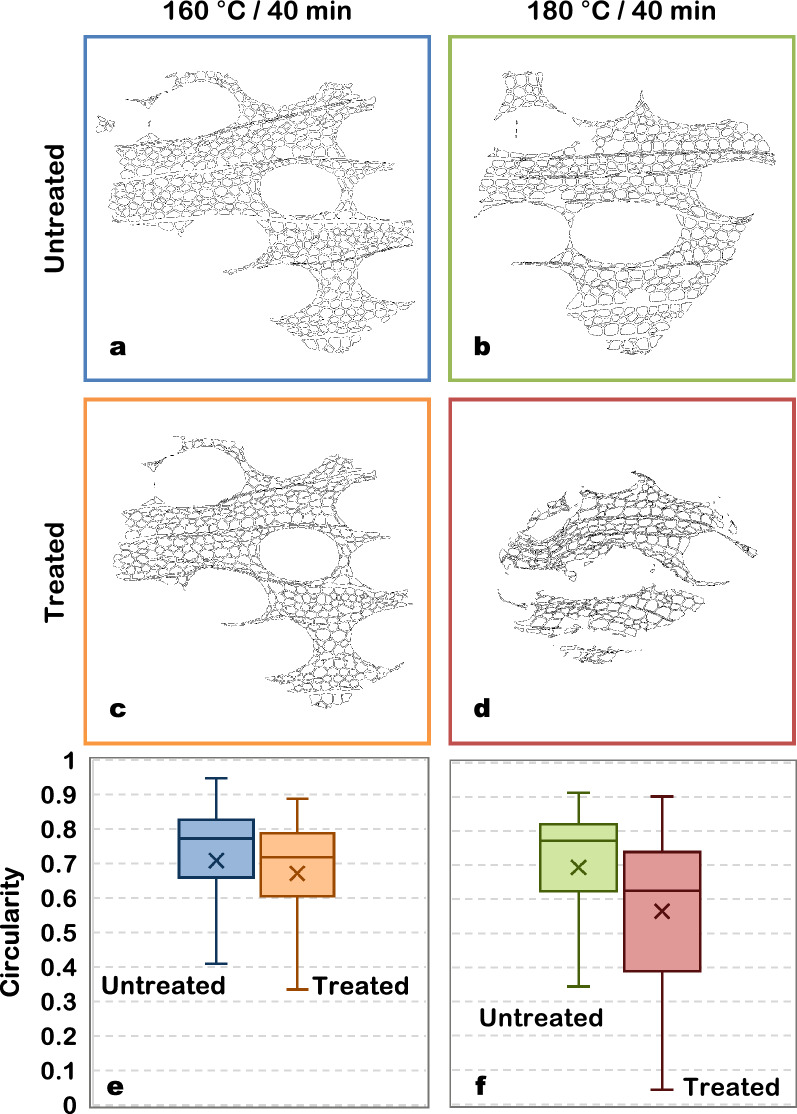
Effect of hydrothermal treatment on the circularity of poplar cells. (**a**–**d**). Automatically identified cell lumens for untreated (**a**,**b**) and hydrothermally treated (**c**,**d**) samples [obtained with the help of ImageJ v1.52i software; imagej.nih.gov/ij/]. (**e**,**f**) Distribution of cell circularity (vessels excluded) of the different samples. The boxes represent the interquartile ranges. Medians and means are represented by the lines inside the boxes and by the markers, respectively. The number of analysed cells was 548 (**a**), 473 (**b**), 408 (**c**) and 254 (**d**).

To further explore the obtained results, X-ray nano-tomography slices were also used to calculate the circularity of the cells (Fig. 7). As expected, no significant difference (*p* value = 0.21) was found between the two samples before treatment (Fig. 7a,b). For the sample treated at 160 °C for 40 min, the mean cell circularity decreased from 0.71 ± 0.18 before treatment (Fig. 7a) to 0.67 ± 0.16 (Fig. 7c) after treatment (*p* value < 0.001). This effect was even more evident for the sample treated at 180 °C (40 min), in which case the mean cell circularity decreased from 0.69 ± 0.19 before treatment (Fig. 7b) to 0.57 ± 0.21 (Fig. 7d) after treatment (*p* value < 0.001). These results support our previous observations. The mass loss suffered by biomass during treatment and the re-organization of cell wall components are likely to induce a fragilization of the cell wall. This loss of mechanical resistance would therefore allow the cells to acquire more elongated forms. Consistently, Zoghlami et al.^11^ demonstrated a reduction in the sphericity of poplar cells with the increase of treatment severity. In the same study, cell sphericity was also found to negatively correlate with the yields obtained after enzymatic hydrolysis. It suggests that, after treatment, samples are more susceptible to the enzymatic attack, probably due to the increased accessibility of the cellulosic fraction.

The shrinkage suffered by the samples due to hydrothermal treatment and subsequent lyophilisation was assessed with the help of MeshPore software^36^ by comparing images of the same sample before and after treatment (Figs. 5c,d and 6c,d). This image analysis revealed a radial shrinkage of 4.1 ± 0.6% and 13.0 ± 2.3% for samples treated for 40 min at 160 and 180 °C, respectively. Shrinkage was higher in the tangential direction, reaching 6.2 ± 1.2% at 160 °C and 21.8 ± 1.8% at 180 °C. This fact is consistent with the above-mentioned reduction of cell circularity. Additionally, tangential shrinkage seems to be more impacted by temperature, as evidenced by an increase in the ratio between tangential and radial (T/R) shrinkage from 1.51 at 160 °C to 1.68 at 180 °C. This anisotropic behaviour, typical of wood, is often observed in the case of wood hygroexpansion and generally attributed to a restrained shrinkage in the radial direction due to the presence of ray tissue^37,38^. Shrinkage as a result of hydrothermal treatment has been observed in a previous work^29^, that used it as a macroscopic indicator of water-saturated biomass degradation during hydrothermal treatment. In the present study, samples subjected to nano-tomography are in a dry state, which exacerbates the already considerable shrinkage and highlights the fragility of treated cells.

**Figure 8:**
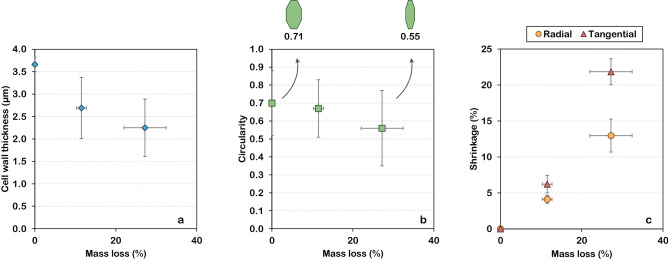
Morphological and physical properties as a function of mass loss. (**a**) Cell wall thickness. (**b**) Circularity, showing cells shapes presenting a circularity of 0.71 and 0.55. (**c**) Shrinkage.

Shrinkage and decrease in cell wall thickness are connected phenomena and can both be linked to mass loss, which reached 11.5 ± 1.2% and 27.2 ± 5.2% after 40 min of hydrothermal treatment at 160 and 180 °C, respectively. This significant loss is logically reflected in sample dimensions. As previously stated, hemicelluloses are the most sensitive components of wood to this kind of treatment, representing the most significant source of mass loss^28,29^. Furthermore, mass loss has been proven to be an excellent indicator of the treatment severity in the case of wood mild pyrolysis^34^ and torrefaction^39^. In this study, the properties measured at different treatment severities were plotted as a function of mass loss (Fig. 8). Even if the curbs only contain three points, some trends emerge from the different properties, confirming the pertinence of mass loss as a macroscopic indicator. Indeed, cell wall thickness and circularity tend to linearly decrease as mass loss increases. Concerning shrinkage, whereas a linear behaviour is observed for the radial direction, it is not observed in tangential shrinkage, which sharply increases with the mass loss. Indeed, for the most severe pre-treatment (180 °C/40 min), shrinkage may also be a result of drastic changes in the biomass anatomy. Figure 6g,h shows that at this intensity level many fibres start to detach from adjacent cells (a few examples are highlighted by the red arrows), seldom seen in the treatment at 160 °C (Fig. 5g,h). The same process can be observed in the SEM images previously shown (Fig. 4c). These observations are consistent with those of previous studies^32,40^. Additionally, cell collapse, which intervenes mainly in the tangential direction, is also one of the major causes of the discrepancy of tangential shrinkage. As shown in Fig. 6g,h, vessels and rays are particularly fragile to this kind of treatment. Cell walls of vessels and rays parenchyma are thinner than those of fibres, which explains their fragility. Vessels have been completely disrupted, as shown by the blue arrows (Fig. 6h) and collapsed.

The hydrolysis of hemicelluloses and the modifications experienced by lignins are likely the main cause of all these structural changes. In untreated wood, hemicelluloses and lignins are responsible for bonding the structure together within the layers of one cell or between neighbour cells^30^. Previous works have found that the hydrolysis of hemicelluloses and the loss or migration of lignins can induce substantial ultra-structural changes on the biomass network^5,20,41^, owing to a loss of mechanical properties and a fragilization of middle lamella. The loss of cohesion between cells and cell wall layers weakens biomass structure and is likely to make it more susceptible to the shearing forces arising during the explosion step. This effect is realized when neighbour cells detach from each other. In this case, the double cell wall of adjacent cells is split in half, meaning that the explosion faces less resistance than from that of the original cell wall.

## Conclusions

Using a unique combination of several imaging techniques this study investigated the effect of hydrothermal treatment of poplar on its chemical composition, ultrastructure and cellular structure. Hemicellulose hydrolysis and lignin migration were observed by confocal Raman microscopy. The chemical changes observed at the cell wall level have later been connected to ultrastructural modifications. Overall, after treatment, samples presented thinner cell walls and significant shrinkage. In more severe treatments, cell disruption and detachment from adjacent cells were also observed. Nano-tomography allowed an overall (3D) vision of biomass degradation after hydrothermal treatment, without any preparation artefacts.

The detachment of neighbor cells and the reduction of cell wall thickness observed in the most severely treated samples enhance the susceptibility to the subsequent explosion phase. Complementary, nano-tomography analyses of intermediate severities would provide database for improved steam explosion modelling, simulation and optimization by linking these indicators of biomass fragilization to mass loss. Other macroscopic properties, as the reduction of cell wall thickness and cell circularity, are both a macroscopic expression of the hydrolysis of hemicelluloses and the delocalization of lignins. These chemical changes may contribute to the accessibility of cellulose by enzymes. The access of enzymes is also facilitated by disruption of some cells, exposing inner portions of biomass.

Finally, an important outcome of this work is that the comprehensive investigation quantitatively linked the effects of treatment at several spatial scales, providing an assessment of the treatment severity based on easily measured macroscopic parameters, such as shrinkage.

## Materials and methods

### Biomass

A 25-year-old poplar tree (*Populus euroamericana* ‘Koster’) from a forest in Auménancourt-le-Petit (France) was used as a source of biomass. The poplar was kindly provided by the Huberlant sawmill (Cormicy, France), who chopped down the tree, cut it into boards and subsequently air-dried the wood. Experimental research and collection of plant material was in accordance with relevant institutional, national and international guidelines and legislation. All samples were obtained from a single defect-free board with a straight grain angle and carefully cut along the longitudinal direction, in which the natural variability of wood is the lowest. The sample size and preparation method depended on the constraints of each imaging technique.

### Hydrothermal treatment

Hydrothermal treatment was conducted in an in-house developed device, described in previous works^21,42^. This device allows temperature to be perfectly controlled by two electric heating collars (370 W) placed at the outer surface of the chamber and a PID device (Eurotherm 3216—Schneider Electric) equipped with a K-thermocouple.

The hydrothermal treatment requires samples to be saturated in water to allow a good heat transfer and reproduce the industrial process, where water vaporization is essential for the explosion step. Samples were saturated in distilled water for 12 h using vacuum-pressure cycles. To avoid any deformation from sudden contact with liquid water, all samples were previously submitted to a pre-saturation step, during which they were kept at 75% relative humidity (RH) conditions overnight.

Treatments were performed at 160 and 180 °C under saturated water vapor conditions. The reaction chamber was heated from room temperature to the treatment temperature (at around 8 °C/min). The chamber was then kept at a constant temperature for 0, 10, 20 or 40 min (plateau duration) and then cooled down (approximately 9 °C/min) to room temperature. It is important to underline that the so-called 0-min treatment means that the chamber was heated to the treatment temperature and then immediately cooled down. After treatment, samples were subjected to different protocols described in the following sections.

In addition to the samples dedicated to chemical and anatomical observations, paired samples were used for determining the moisture content of native samples and for assessing the mass loss at each treatment condition. The initial moisture content was assumed constant throughout the samples and its value was used to correct the initial mass of each sample to its corresponding dry mass. The mass of the treated samples was determined after oven-drying at 105 °C for 12 h. The mass loss (ML) induced by hydrothermal treatment was calculated according to the following equation:

1 $$ ML{ }\left( {\text{\% }} \right) = { }\frac{{M_{0} - M}}{{M_{0} }}\times{ }100 $$

where *M*_0_ is the initial dry mass of the sample and *M* is its dry mass after treatment. Three repetitions were made for each treatment condition. The ML values obtained were 11.5 ± 1.2% and 27.2 ± 5.2% after 40 min of treatment at 160 °C and 180 °C, respectively.

### Poplar compositional analysis

The amounts of cellulose, hemicellulose, acid soluble and insoluble lignins were determined (in triplicate) for the native biomass and the solid fraction of hydrothermally treated samples. The methodology was based on the National Renewable Energy Laboratory (NREL) standard procedure^43^. The standard procedure had to be adapted to some particularities of our experimental set-up. All changes have been reported in a previous work^29^. The results presented in Fig. 3 are ratios between the amount of a compound in the treated biomass and its amount in the native biomass. Dry matter loss due to treatment—reported in the precedent section—is taken into account in the calculations. Native poplar presented the following composition: 45.8 ± 1.9% of cellulose (expressed in terms of glucan), 20.6 ± 0.8% of hemicellulose (expressed in terms of xylan), 20.7 ± 3.9% of insoluble lignins and 8.3 ± 0.5% of soluble lignins.

### Confocal Raman microscopy

#### Sample preparation

Poplar cubes (8^3^ mm^3^) were hydrothermally treated according to the previously described protocol. Treated water-saturated samples were stored in a closed chamber at 75% relative humidity for two days to allow slow drying and to minimize deformation and cell wall collapse. Samples were then dried in ambient air for two more days. Control and hydrothermally treated 3 × 3 × 8 mm poplar blocks were then split from original cubes along the grain (respectively in radial, tangential and longitudinal directions) and 16 µm thick cross-sectional slices were cut from these blocks using a sliding microtome (Microm HM 450—Thermo Fisher Scientific). Slices were sequentially washed with distilled water, a diluted NaClO solution (0.02% m/v) and distilled water. Washed samples were placed between a microscope slide and a coverslip with a drop of water, to prevent the sample from burning. The set was sealed with nail polish to avoid water evaporation^44^.

#### Image acquisition

Chemical maps of wood fibres were obtained using an Alpha 300R + confocal Raman microscope (Witec), equipped with an objective from Nikon (Plan Apo VC 60 × NA = 1.40 Oil), a piezoelectric x–y stage and a CCD detector. Scans were run at least in triplicate for each combination of treatment temperature and residence time. For each analysed point of the sample (every 0.2 µm), a spectrum was acquired with a 532 nm laser at 10 mW and an integration time of 0.1 s per pixel. Laser-induced fluorescence (LIF) is a known challenge when dealing with Raman spectroscopy of lignocellulosic materials, especially in highly lignified or treated samples^45^. Even though there are no studies showing the application of photobleaching to treated wood, it has been largely used to reduce fluorescence of biological samples, e. g. carotenoids in human skin^46^. At the same time, studies of untreated wood show that Raman bands were not significantly affected by a 10 mW green laser exposure (532 nm), even after repeated measurements, when integration time was less than 0.13 s^47^. In the present study, the application of a photobleaching before spectra acquisition allowed for an increase in the signal-to-noise ratio, as a consequence of fluorescence reduction.

Spectra were treated for cosmic ray removal and baseline correction using WITec’s software Project FOUR. The same software was used for generating chemical maps with a univariate approach, by integration over selected wavenumber bands; and with a multivariate approach, by cluster analysis.

### X-ray nano-tomography

#### Sample preparation

Samples for nano-tomography analysis should be very small to ensure good resolution. However, after pre-treatment, samples are fragile, making sample manipulation challenging. Small samples are also difficult to attach to the sample holder. For these reasons, untreated samples were carved out of wood blocks of 1 cm high in a conical shape. The smaller portion (with a diameter of about 1 mm) was scanned while the larger one (with a diameter of about 2 mm) was used to hold the sample.

To study the effect of hydrothermal treatment on the anatomical structure of biomass, the same samples were scanned before and after treatment. Thus, untreated samples were scanned, then submitted to hydrothermal treatment according to the protocol previously described, and finally re-scanned. To prevent the collapse of treated water-saturated samples, after treatment, these samples were immediately frozen at − 80 °C and later freeze-dried (Alpha 1–2 LD, Martin Christ), as recommended by Donohoe et al.^13^. Preliminary tests were made on untreated samples to evaluate the effect of freeze-drying on anatomical structure (Supplementary Fig. S1). It proved that this methodology does not affect samples morphology.

#### Image acquisition and treatment

Samples were scanned (EasyTomXL Ultra 150–160, RX Solutions) with a voxel size of 500 nm^3^ using a nano-focus vacuum tube for X-ray emission at a working voltage of 100 kV and working current of 170 µA. A total of 1440 radiographs were obtained, each with an exposure time of 2 s and an average of 5 frames, using a CCD detector of 2016 × 1344 pixels. Avizo 2019.2 software (Thermo Scientific) was used to generate 2D cross-sectional slices. For each sample, five slices equally distributed throughout the longitudinal direction were treated with ImageJ software (version v1.52i) to remove the background (Supplementary Fig. S2) and were later analysed for cell wall thickness using the Local Thickness plugin^48,49^ and for circularity using the particle analysis plugin, according to the following equation:

2 $$Circularity = \frac{4\pi A}{{P^{2} }}$$

In which A is the area and P is the perimeter of the identified cell lumens.

The analysis of shrinkage was obtained by comparison of 2D cross-sectional slices of the same sample before and after treatment using the MeshPore software^36^.

### Scanning electron microscopy

After submitted to nano-tomography, the nanostructure of treated samples was observed using scanning electron microscopes. To observe the effect of hydrothermal treatment on the anatomical structure, untreated control samples were also observed. In both cases, the samples were preliminarily freeze-dried.

To study the middle lamella of cells, samples were cross-sectioned using a microtome blade. Because wood is non-conductive, samples were first coated with gold. Observation was made using an environmental scanning electron microscope (ESEM, FEI Quanta 200) using the following conditions: secondary electron detector, high vacuum mode, an accelerating voltage of 10 kV (control sample) and 12 kV (treated sample), a spot size of 3 and a work distance of approximately 9 mm.

To examine the structure of cell walls to a greater depth, a field emission scanning electron microscope (FEG-SEM) Model LEO 1530 (Leo Electron Microscopy Ltd, Zeiss, Oberkochen, Germany) was used. Samples were first split along the longitudinal-radial plane. Then, the non-conductive samples were coated with tungsten (Q150TS, Quorum Technologies). The operating conditions of FEG-SEM were an accelerating voltage of 2 kV, a diaphragm of 30 μm and a work distance of 2 mm.

The images generated by FEG-SEM were later used to measure the size of the droplets. A total of 89 droplets were measured with the help of the ImageJ software (version v1.52i).

## Supplementary Information

Supplementary Information
