# Supplementary material for: Multiscale investigation on the chemical and anatomical changes of lignocellulosic biomass for different severities of hydrothermal treatment
Source: Sci Rep. 2021 Apr 19;11:8444. doi: 10.1038/s41598-021-87928-y (PMC8055998; doi:10.1038/s41598-021-87928-y)
Supplement: Supplementary file 1 — Supplementary Information [file 41598_2021_87928_MOESM1_ESM.docx]

Multiscale investigation on the chemical and anatomical changes of lignocellulosic biomass for different severities of hydrothermal treatment

Julia P. Lancha^1^, Patrick Perré^1,2^, Julien Colin^1,2^, Pin Lv^1^, Nathalie Ruscassier^2^, Giana Almeida^3*^

^1^ Université Paris-Saclay, CentraleSupélec, Laboratoire de Génie des Procédés et Matériaux, SFR Condorcet FR CNRS 3417, Centre Européen de Biotechnologie et de Bioéconomie (CEBB), 51110 Pomacle, France

^2^ Université Paris-Saclay, CentraleSupélec, Laboratoire de Génie des Procédés et Matériaux, 8-10 rue Joliot-Curie, 91190 Gif-sur-Yvette, France

^3^ Université Paris-Saclay, INRAE, AgroParisTech, UMR SayFood, 91300 Massy, France

*giana.almeida@agroparistech.fr

**Supplementary Information:**

**Figure S1.** Preliminary tests performed to choose the sample preparation method prior to X-ray nano-tomography. Two samples have been scanned in their native state (air-dry state, Figures S1a and S1b). They were then saturated with water and hydrothermally treated at 180 °C for 40 minutes. After treatment, sample 1 was left under room conditions (air drying) for two days, whereas sample 2 was frozen at -80 °C and then freeze dried before returning to the air-dry state. Both samples were later re-scanned (Figures S1c and S1d, for samples 1 and 2 respectively). Sample 1 (Figure S1c) suffered extensive shrinkage and many of its cells collapsed after air drying. Sample 2 (Figure S2d) presented less shrinkage and only minor cell collapse (which was limited to the cells located between two vessels). The dashed lines highlight the changes in samples dimensions.

**
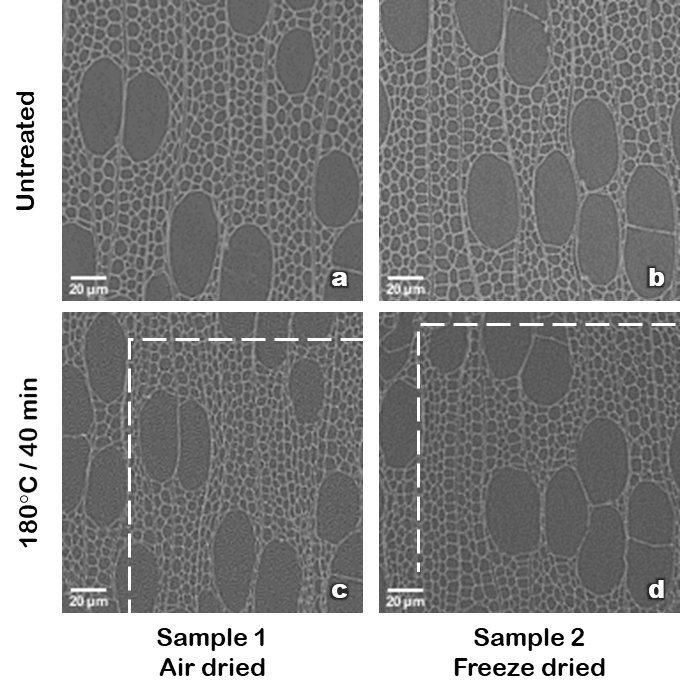
**

**Figure S2.** Steps of image processing using Image J applied to 2D cross-sectional slices obtained by X-ray nano-tomography: (a) Raw image; (b) Image after segmentation by the “Auto Threshold” tool using the “Moments” method for dark background; (c) Mask obtained using the “Particle analysis” tool, with a minimum particle size set to 5000 pixel²; (d) Final image after “Local thickness” analysis.

**
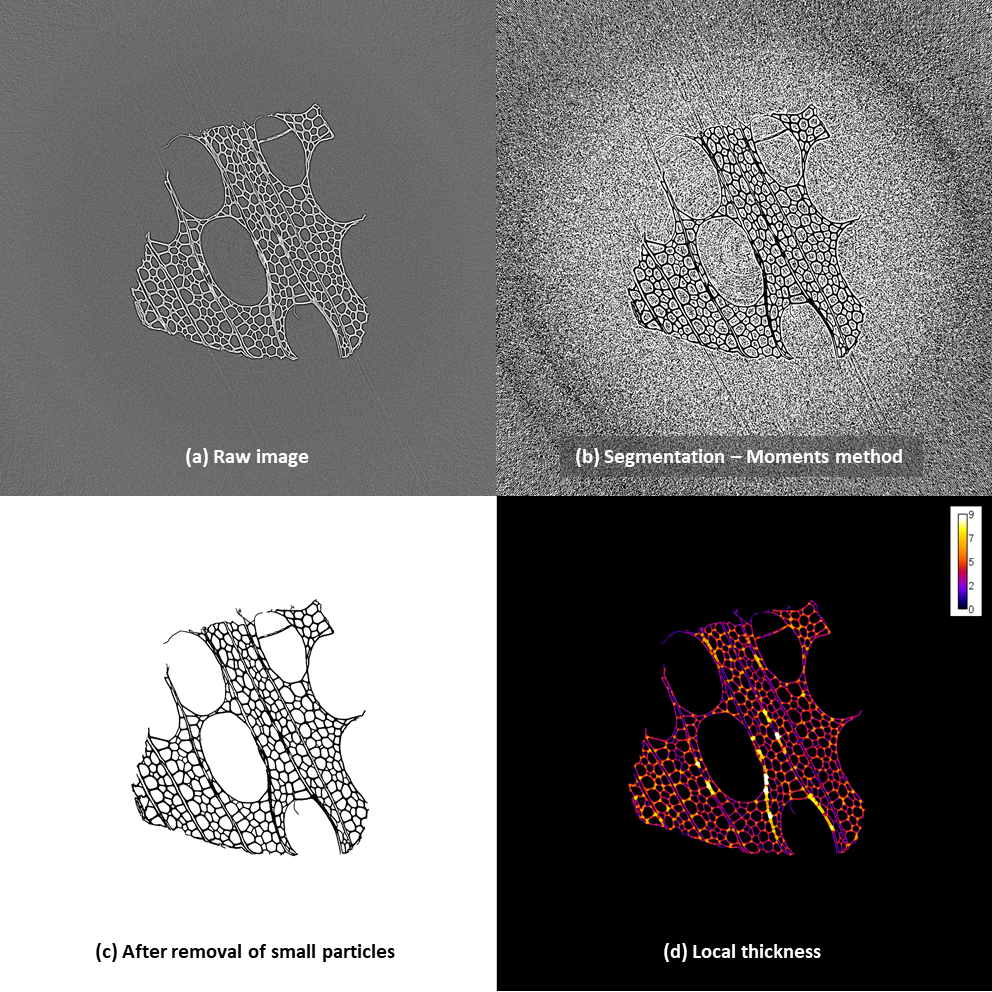
**
